# Supplementary material for: Prediction of coronary heart disease in rural Chinese adults: a cross sectional study
Source: PeerJ. 2021 Oct 11;9:e12259. doi: 10.7717/peerj.12259 (PMC8515995; doi:10.7717/peerj.12259)
Supplement: Supplemental Information 1 — 1Data were shown as number of samples, the regression results were shown as odds ratios (95% confidence interval) and P values. 2N1: Number of samples in CHD group. N2: Number of samples in non-CHD group. [file peerj-09-12259-s001.pdf]

Supplementary table 1. Univariate logistic regression of all variables and the outcome variable (CHD and non-CHD).

Variable[1]	N1/N2[2]	OR (95% CI)	P
Gender			
Male	346/433	Reference	
Female	507/1025	0.62 (0.52-0.74)	<0.001
Age		1.15 (1.14-1.16)	<0.001
Smoking			
Never	608/1201	Reference	
Current	110/220	0.99 (0.77-1.27)	0.922
Ever	135/37	7.21 (4.95-10.50)	<0.001
Drinking			
Never	662/1210	Reference	
Current	109/227	0.88 (0.69-1.12)	0.301
Ever	82/21	7.14 (4.38-11.63)	<0.001
Pressure			
No	656/846	Reference	
Slight	90/313	0.37 (0.29-0.48)	<0.001
Moderate	40/187	0.28 (0.19-0.39)	<0.001
Severe	55/89	0.80 (0.56-1.13)	0.205
Extreme	12/22	0.70 (0.35-1.43)	0.332
PSQI		1.27 (1.22-1.31)	<0.001
BFR		1.16 (1.14-1.18)	<0.001
BMR		1.00 (1.00-1.00)	<0.001
VFI		1.33 (1.30-1.37)	<0.001
SBP		1.11 (1.10-1.12)	<0.001
DBP		1.12 (1.11-1.13)	<0.001
WHR		6.20E+07 (1.26E+07-3.05E+08)	<0.001
BMI		1.07 (1.04-1.09)	<0.001
WBC($\times 10^9$/L)		1.23 (1.16-1.30)	<0.001
PLCR%		0.99 (0.98-1.00)	0.203
MONO%		0.94 (0.88-0.99)	0.028
MONO($\times 10^9$/L)		5.40 (2.47-11.80)	<0.001
HCT		1.10 (1.08-1.12)	<0.001
RDW_CV		0.99 (0.93-1.06)	0.862
RBC($\times 10^{12}$/L)		1.65 (1.36-2.00)	<0.001
RDW_SD		1.07 (1.05-1.09)	<0.001
LYMPH%		1.00 (0.99-1.01)	0.503
LYMPH($\times 10^9$/L)		1.60 (1.38-1.86)	<0.001
MCV		1.05 (1.04-1.07)	<0.001
MCH		1.13 (1.08-1.17)	<0.001
HCHC		1.00 (1.00-1.01)	0.559

MPV		0.95 (0.87-1.04)	0.244
BASO%		1.32 (0.99-1.78)	0.06
BASO($\times 10^9$/L)		8.00E+04 (5.18E+02-1.24E+07)	<0.001
EOS%		1.07 (1.02-1.12)	0.003
EOS($\times 10^9$/L)		5.82 (2.72-12.43)	<0.001
HGB		1.02 (1.02-1.03)	<0.001
PCT%		0.08 (0.02-0.32)	<0.001
PDW		0.99 (0.95-1.03)	0.743
PLT($\times 10^9$/L)		1.00 (1.00-1.00)	0.007
NEUT%		1.00 (0.99-1.01)	0.807
NEUT		1.20 (1.12-1.29)	<0.001
LDL_C		5.99 (4.98-7.20)	<0.001
TG		20.16 (15.24-26.68)	<0.001
HDL_C		0.10 (0.07-0.14)	<0.001
TC		5.23 (4.43-6.16)	<0.001
FBG		3.39 (2.91-3.95)	<0.001
HbA1c		8.01 (6.47-9.91)	<0.001
FINS		1.11 (1.08-1.13)	<0.001
ALT		1.03 (1.02-1.03)	<0.001
Creatinine		1.04 (1.03-1.05)	<0.001
IBIL		1.03 (1.02-1.05)	<0.001
ALP		1.03 (1.02-1.03)	<0.001
Urea		1.37 (1.29-1.46)	<0.001
Uric_acid		1.01 (1.00-1.01)	<0.001
AST		1.04 (1.03-1.05)	<0.001
DBIL		1.00 (0.95-1.06)	0.952
TBIL		1.02 (1.01-1.03)	<0.001
THB		1.08 (1.05-1.11)	<0.001
HR		1.00 (1.00-1.01)	0.275
RR		1.08 (0.57-2.05)	0.816
PR		19.91 (0.81-490.53)	0.067
QRS		1.74E+07 (1.72E+04-1.76E+10)	<0.001
QT		2.40E+04 (1.21E+03-4.76E+05)	<0.001
QTC		8.84E+09 (1.41E+08-5.56E+12)	<0.001
SV1+RV5		1.57 (1.40-1.76)	<0.001
ECGNOTE			
Normal	478/1305	Reference	
Load	143/102	3.83 (2.91-5.04)	<0.001
Overload	231/51	12.37 (8.97-17.04)	<0.001

[1] Data were shown as number of samples, the regression results were shown as odds ratios (95% confidence interval) and P values.

[2] N1: Number of samples in CHD group. N2: Number of samples in non-CHD group.
